# Recent Trends and Perspectives in Cerebral Organoids Imaging and Analysis

**DOI:** 10.3389/fnins.2021.629067

**Published:** 2021-07-02

**Authors:** Clara Brémond Martin, Camille Simon Chane, Cédric Clouchoux, Aymeric Histace

**Affiliations:** ^1^ETIS Laboratory UMR 8051, CY Cergy Paris Université, ENSEA, CNRS, Cergy, France; ^2^WITSEE, Paris, France

**Keywords:** image analysis, microscopy, 3D brain cultures, organoid, morphology

## Abstract

**Purpose:** Since their first generation in 2013, the use of cerebral organoids has spread exponentially. Today, the amount of generated data is becoming challenging to analyze manually. This review aims to overview the current image acquisition methods and to subsequently identify the needs in image analysis tools for cerebral organoids.

**Methods:** To address this question, we went through all recent articles published on the subject and annotated the protocols, acquisition methods, and algorithms used.

**Results:** Over the investigated period of time, confocal microscopy and bright-field microscopy were the most used acquisition techniques. Cell counting, the most common task, is performed in 20% of the articles and area; around 12% of articles calculate morphological parameters. Image analysis on cerebral organoids is performed in majority using ImageJ software (around 52%) and Matlab language (4%). Treatments remain mostly semi-automatic. We highlight the limitations encountered in image analysis in the cerebral organoid field and suggest possible solutions and implementations to develop.

**Conclusions:** In addition to providing an overview of cerebral organoids cultures and imaging, this work highlights the need to improve the existing image analysis methods for such images and the need for specific analysis tools. These solutions could specifically help to monitor the growth of future standardized cerebral organoids.

## 1. Introduction

### 1.1. Historical Context

Experimental cerebral models are used to observe and analyze structure and function, both of which are complex to identify in human brain tissues (Stan et al., 2006). These models are often classified in three categories: *in vivo*, post-mortem, and *in vitro*. However, *in vivo* and post-mortem brain animal models are often prone to controversy due to ethical considerations added to technical impairments due to divergences with the human brain structures (Lodato et al., 2015; Kelava and Lancaster, 2016a). Key benefits of *in vitro* models are that these cultures can be derivatives from human cells, on the one hand, and, on the other hand, be more relevant to replicate its physiology. Despite these benefits, standard 2D neuronal cultures lack of tissue structures, diversity of self-patterning cells and some disease patterns, presenting then with strong limitations for *in vitro* study. Three-dimensional (3D) brain cultures (Kapalczynska et al., 2016; Bolognin et al., 2019; Cederquist et al., 2019) have become in the last years a very promising alternative to overcome these limitations.

In this context, recently, cerebral organoids (CO) have emerged by the differentiation of reprogrammed pluripotent stem cells (iPSCs), or human embryonic stem cells (hESCs) (Lancaster et al., 2013). Such 3D cultures are no larger than 4 mm in diameter and they develop some structures similar to those developed by the brain during the second semester at numerous random locations (Kelava and Lancaster, 2016b). To study these cerebral organoids, researchers use methods originally developed to analyze other post-mortem and *in vitro* models: enzyme-linked immunosorbent assay (ELISA Raja et al., 2016), quantitative retrotranscriptase-polymerase chain reaction (RTqPCR Sakaguchi et al., 2015), ribonucleic acid sequencing (RNAseq Quadrato et al., 2017), micro-electrode array (MEA Monzel et al., 2017), and others techniques focused on, for example, proteins or metabolites. Because these techniques can lead to complex and costly experimental set-up, in addition to them, imaging techniques are now used in almost every study focusing on cerebral organoids both to complete and to validate other molecular analysis. It can also be used to observe features that are unavailable with other methods, for example to quantify the growth of such cerebral organoids (Iefremova et al., 2017).

**Figure 1:**
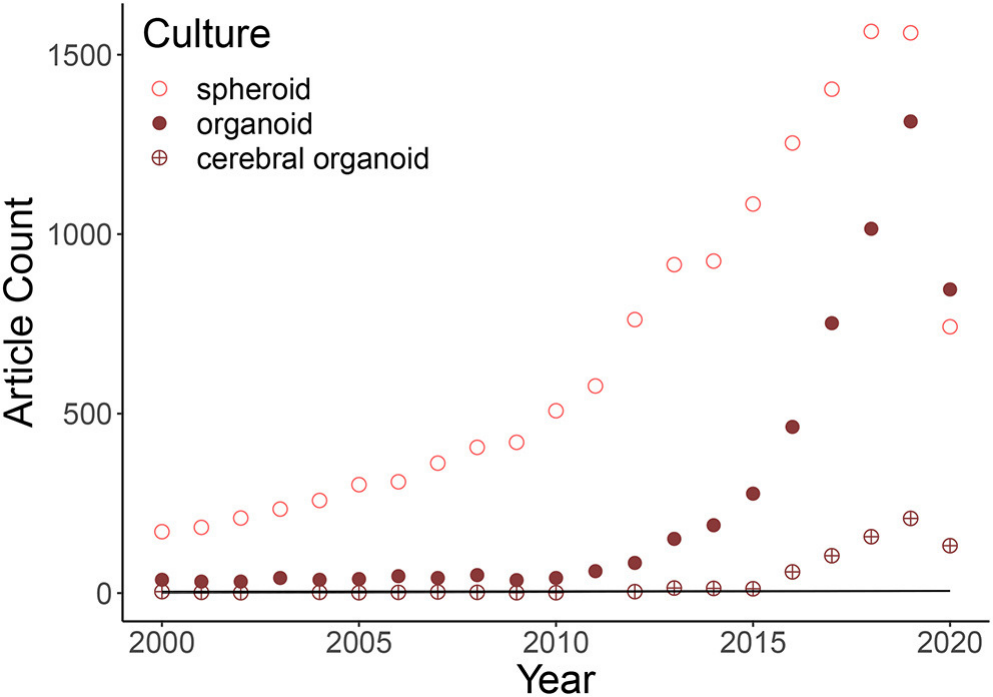
Graphic representation of the soaring of three-dimensional cultures based on a pubmed search of the following keywords: “spheroid,” “organoid,” and “cerebral organoid.” Cerebral organoid articles are a subset of the keyword “organoid” research. The expanding of published articles is explained by an exponential model at 89% (Rsquared: 0.8876: Growth Model = log(Count) Year:Culture). The first generation of cerebral organoids was in 2013, so the previous few articles identified by pubmed contain the two keywords but do not talk about these 3D brain cultures inside the body of the text. The points in 2020 are not on the curves due to the fact that the year is not over.

The commercialization of cerebral organoids since 2016 (Chakradhar, 2016) has resulted in the widespread generalization of their use by laboratories (see Figure 1). Consequently, the microscope technique, analysis methods, and tools must be tailored for the issue at hand.

Given this increase and the importance of the image analysis in this field, it has become essential to identify the methods employed to study cerebral organoids, as well as the improvements that can be performed and the challenges that need to be overcome to handle image analysis on cerebral organoids at a larger scale.

### 1.2. Scope and Positioning of This Review

This review summarizes the recent advances in 3D brain cultures imaging and analysis, and particularly for cerebral organoids. We performed statistical analysis on the 457 articles on cerebral organoids referenced by Pubmed between January 2018 and June 2020. We chose to perform this review study starting from 2018 because the number of articles per year was <100 before this date. Of note, 670 articles on key words “cerebral organoids” have been published since 2013 according to Pubmed. Among these 457 articles, 63 mentioned these key words but are not on this topic, and 46% of the remaining articles are reviews.

**Figure 2:**
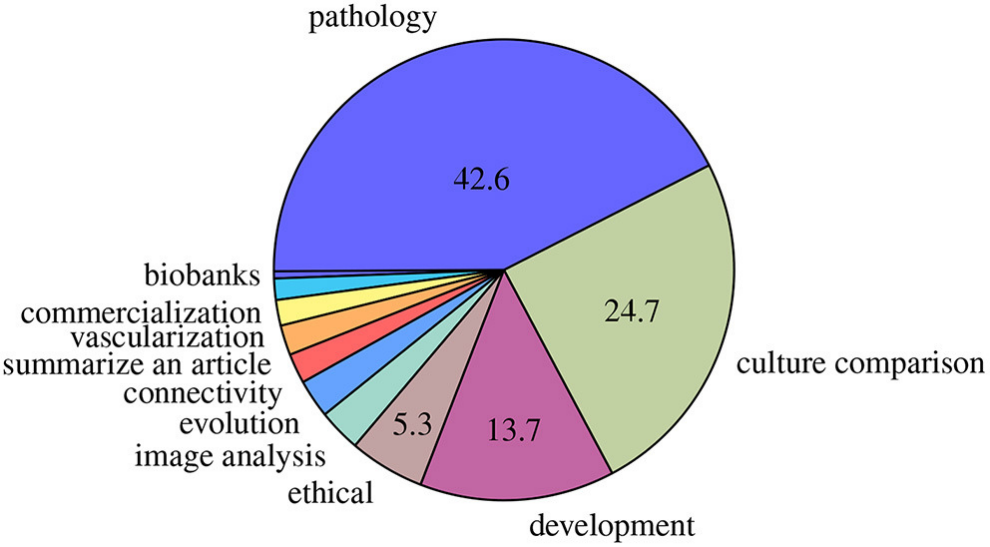
Main domains of reviews published. Percentages <5% are not mentioned.

Most of these reviews addressed brain diseases, cultures comparisons including a review on the possible emergence of cerebral organoids connected to other organ models (Chukwurah et al., 2019), and development (Figure 2). Less than 3% of the reviews addressed 3D brain cultures images analysis. Among them, only three about image analysis applied to cerebral organoids data have been published. Poli et al. (2019) reviewed computational models of formation and organization of these cultures, and also reviewed protocols and other experimental methods (in electrophysiologic field) applied on cerebral organoids. For these authors, even if cerebral organoids are promising in terms of *in vitro* models of human brain, the generation protocols and procedures characterization still need refinement. Booij et al. (2019) analyzed imaging techniques, image analysis methods and high-content images in 3D cultures but not particularly focused on cerebral organoid cultures. They concluded on the requirement to "validate these technologies and to demonstrate clearly that using biologically relevant *in vitro* systems actually improves the efficiency of early drug discovery. A direct comparison of the predictive value of 2D and 3D models for *in vivo* efficacy is required.” Grenier et al. (2020) mentioned in a diagram the perspective of generating a high-throughput platform for drug testing including image analysis on cleared cerebral organoids with deep learning to identify functional and architectural markers. The authors also discussed the challenges allowing integration of additional variables and risk factors (toxic agents, vasculature) in order to make cerebral organoids a formidable and scalable system to improve our understanding, provide precision to diagnostic and prognostic predictions and personalize drug discovery efforts for neurodegenerative diseases Of note, in another field, Boutin et al. (2019) studied retinal organoids to summarize perspectives on drug testing. One of their expectations was also to apply machine learning on both high-content cell imaging and others chemical methods for their retinal model. They expected work was "being done to apply machine learning approaches to score and predict control vs. disease phenotypes from cell imaging assays, including work on photoreceptor outer segment formation. Most of this work has so far been done in 2D systems, and the hope is that with the development of techniques that allow HT cell imaging in 3D, those will be applied to this more complex systems.”

In the time range considered for our paper, we did not find any review focused on image analysis tools dedicated to cerebral organoids. However, a very recent study was published by Albanese et al. (2020) (December), creating for the first time a pipeline named Scout including deep-learning methods to segment the ventricular zone of 3D images of cleared cerebral organoids. They gave a first attempt to a holistic approach to characterize the content and structure of cerebral organoids in 3D.

The current review focuses on the recent trends in acquisition and image analysis methods on cerebral organoids to highlight the specific needs of the field. For all 214 included articles published on cerebral organoids, between January 2018 and July 2020, we identified their scope; the kind of organoid generated; the acquisition method of images presented in the figures; the analysis methods used specifically, the software and algorithms developed or used; and finally advantages and limitations of the proposed approaches.

The following section gives an overview of the emergence of 3D brain cultures. Then we describe the sample preparation and the image acquisition methods. Three-dimensional imaging is particularly detailed in this paragraph because it captures better the shape and allows quantification for the full brain culture. In the third section, image analysis methodologies are described in two parts: quantification and morphological analysis. Software used for that particular aim are presented in the fourth section. They remains for the most part semi-automatic due to the recent generation of this model. Following this methodology section, we discuss the pros and cons of each described method, as well as the potentially insightful image analysis tools to implement in order to handle the increasing amount of generated data.

## 2. Three-Dimensional Brain Cultures

### 2.1. Advent of Cerebral Organoids

Over the past 10 years, a considerable increase in the use of 3D cultures has been observed. Figure 1 shows an exponential growth in the number of articles citing spheroids, organoids, and cerebral organoids. Between 2013 and June 2020, 671 out of 4509 published articles on organoid cultures were treating about cerebral organoids. Before explaining how imaging cerebral organoids, we summarized in this section what are cerebral organoids and how their generation has evolved in the last decade.

Organoids mimic organs: they contain multiple organ-specific cell types, are spatially organized, and simulate organ-specific functions (Lancaster and Knoblich, 2014b).

The first 3D neural organoid was a self-organized optic cup made of retinal epithelium (Eiraku et al., 2011). Two years later, Kadoshima et al. (2013) created guided forebrain organoids and Lancaster et al. (2013) the first self-patterned cerebral organoids. These organoids replicate human fetal brain growth during the second semester (Kelava and Lancaster, 2016b). The discrepancy between these two cultures is mainly due to the growth pattern and both methods are currently used for cerebral organoid generation.

**Figure 3:**
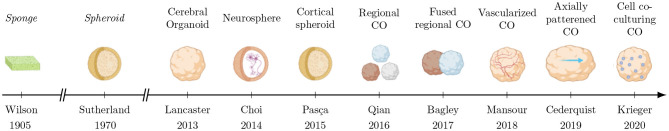
Evolution of 3D brain cultures over time. Non-brain cultures which led the way are labeled in italics. The abbreviation CO is used for “cerebral organoid”.

Pasca et al. (2015) created cortical spheroids, also called dorsal forebrain organoid (Arlotta and Pasca, 2019), an assembly of differentiated cells producing deep and superficial layers around ventricular zones. Then, 3D bio-printing bioreactors allowing the generation of cerebral region-specific organoids (forebrain, midbrain and hyppocampic) have emerged (Qian et al., 2016). While these region-specific have been created, some authors proposed to fuse them to reproduce the connectivity observed between structures in the human brain (Bagley et al., 2017; Birey et al., 2017). One of the remaining weaknesses of this system is the absence of vasculature, later Mansour et al. (2018) transplant cerebral organoids inside *in vivo* model to vascularize the culture. Others teams observed that human organoid transplantation inside injured *in vivo* mice brains helped lost functions recovering (Wang et al., 2019). Nevertheless, the inter-organoid heterogeneity and their cell diversity, failing to reproduce the topological organization of the human brain, conduct others authors to axially pattern cerebral organoids as occurring during the fetal growth (Cederquist et al., 2019). Only recently, cerebral organoids have been co-cultured with others cell type (tumoral for example), to model disease progression (Krieger et al., 2020). Figure 3 summarizes the evolution of 3D cultures from sponges to modern cerebral organoids.

### 2.2. Variability in 3D Brain Cultures

The importance of imaging cerebral organoids is linked to their particular constitution. The cyto-architectural complexity of cultures mimicking brain formation (see Figure 3) greatly depends on the culture protocol (Sidhaye and Knoblich, 2020). Cerebral organoids containing self-patterned regions are larger and more complex than cortical spheroids showing rosette patterns. In turn, these are more complex than an assembly of different cell-types in a neurosphere (Kelava and Lancaster, 2016b). However, differentiating a regional cerebral organoid (i.e., dorsal or ventral forebrain) is more tedious than letting a cerebral organoid self pattern, as such differentiation requires various factors additions to the media at specific times (Lancaster et al., 2013; Bagley et al., 2017).

**Figure 4:**
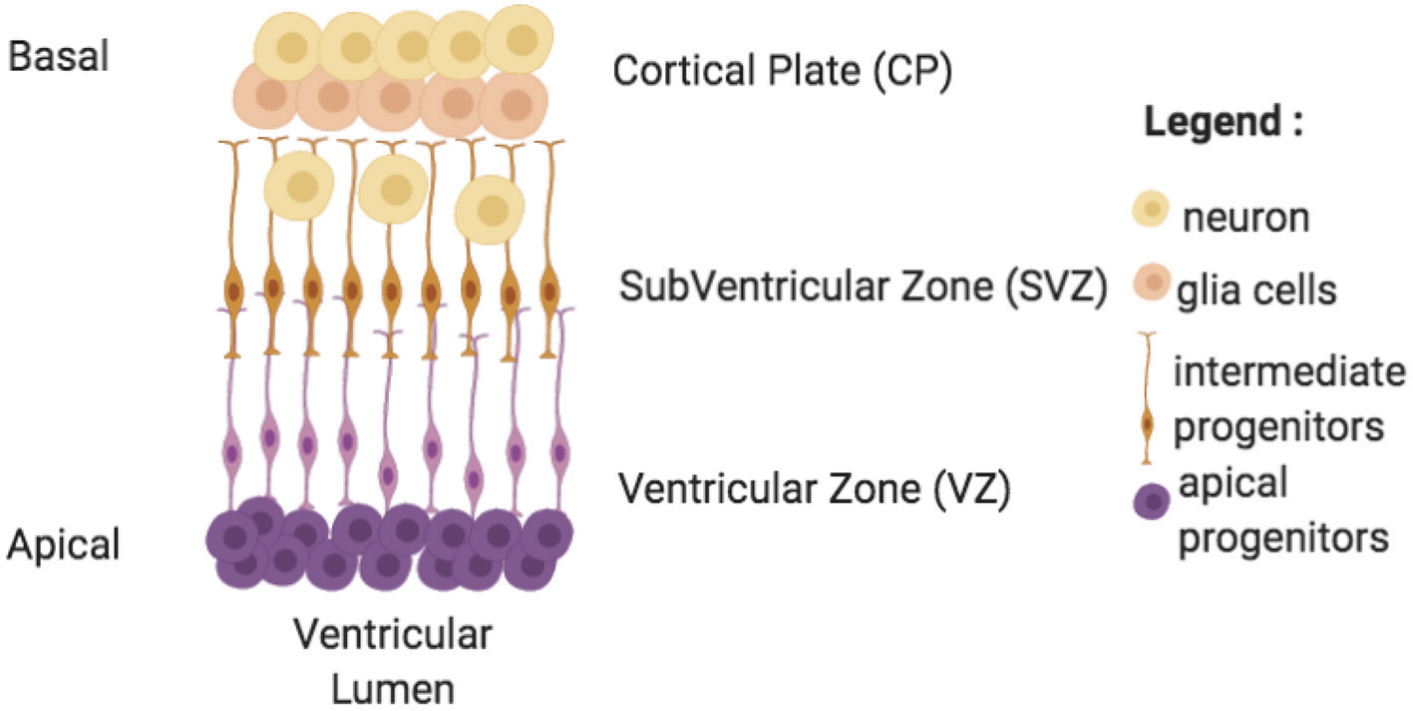
Neuroepithelium formation inside cerebral organoids. This formation is present at discrete random locations around lumen ventricles in cerebral organoids. It grows from the apical perimeter thanks to progenitor cells to the basal zone. Cells migrate and differentiate along these axes. Cerebral organoids' neuroepithelium are made of 3 zones: a ventricular zone (VZ), a subventricular zone (SVZ), and a cortical plate (CP) each composed of specific cell types.

During the cerebral organoid generation process, model complexity increases with time. First, iPSCs are derived and aggregated in an embryoid body, which undergoes a neural induction (containing a core and a peripheral zone). It is then embedded in a matrix for maturation (Kelava and Lancaster, 2016b). During the maturation phase, cerebral organoids innately almost mimic second semester fetal brain growth by developing neuroepithelium regions (Figure 4; Lancaster and Knoblich, 2014a). Similarly to human development, neuroepithelium are constituted by a ventricular zone surrounding lumen, a subventricular zone (more recently, both inner and outer subventricular zone were generated Qian et al., 2020) and a cortical plate constituted by various cell populations with neurons producing action potentials and synapses (Lancaster et al., 2013). Moreover, comparative studies between fetal human brain developmental stage and cerebral organoids showed some similar transcriptome even if few genes are down or up regulated (Qian et al., 2016). However, there are more complex signatures in the human case due in part to vascularization, to radial glia frequency, and to consequent neuron generation in later fetal stages (Qian et al., 2016; Bershteyn et al., 2017). Despite these differences and different growth conditions, parallels can potentially be made between human brain and cerebral organoid tissues development, as investigated in some studies, using histological images (LaMonica et al., 2013; Ostrem et al., 2015; Kostovic et al., 2019).

Time and growth are also quite important parameters, since they can lead to necroses at the core of cultures, mostly due to shortage in nutrients and oxygenation. An answer to this problem consists of slicing cerebral organoids during their growth (Qian et al., 2020). Such a process increases the number of neuroepithelium layers and the culture longevity.

An important morphological variability between cultures of the different batches exists ("batch to batch syndrome"), as well as variability within a given batch ("batch syndrome"), although not as important as the former. Such variability consists of regions developing in various locations and in an undetermined number (Lancaster et al., 2013). One explanation lies in the non-homogenization between pluripotent stem cells at the origin of the cerebral organoid colonies in term of morphology and pluripotency. Another reason is the thickness of media culturing (Poli et al., 2019). Such variability precludes atlas creation for cerebral organoids (Zaslavsky et al., 2014). In order to reproduce the brain cyto-architectural development with a higher reproducibility, some studies investigated the addition of specific factor to the media (WNT, SSH, FGF)(Krefft et al., 2018; Cederquist et al., 2019; Kim et al., 2019; Sivitilli et al., 2020), whereas others used bioreactors (Qian et al., 2016; Eremeev et al., 2019; Velasco et al., 2019) or changed the type of culture (Berger et al., 2018; Nickels et al., 2020).

Many authors also chose to study cerebral organoids replicating various diseases (neuro-developmental, neuro-degenerative, tumoral, infectious or injury models) originating from patient biopsies (Tian et al., 2020). Indeed, almost the half of the reviewed articles studies cerebral organoids model disease (Table 1). Cerebral organoids are complex to produce and standardize, but they are already used in pathological cases. The complexity of studying cerebral organoids is also related to protocols and imaging methods described in the following section.

**Table 1:** Percentage of articles studying diseases on cerebral organoids over 2019 and 2020.

**Organoid model**	**Articles (%)**
Healthy	52.34
Neurodevelopmental	14.95
Neurodegenerative	11.68
Tumor	8.41
Infection	8.41
Injury	3.74

*Neurodegenerative diseases include articles on Alzheimer and Parkinson. Neurodevelopmental diseases include autism, lissencephaly, microcephaly, skizophrenia, and various syndromes. Tumors include glioblastoma invasion in cerebral organoids. Infections correspond to viral infections, and injury to brain lesions*.

### 2.3. Microscopic Studies of 3D Brain Cultures

Cerebral organoids and other 3D brain cultures are studied both as a whole and at the molecular, cellular, or regional level. The frequencies, aims, and major disadvantages are summarized in Table 2 Cerebral organoids are most often studied by microscopic observation and analysis. A small fraction of articles do not use microscopy: these either propose a new model or they only rely on RNAseq for the analysis. Cerebral organoids are generally studied first intact and then sliced, as shown in Figure 2. A few studies (4.33%) study whole clarified organoids. Most studies produce fluorescent images from confocal microscopy (54.53%). The two main analysis performed on these images of 3D cultures are quantifications (counting cells and their components, measuring marker intensity or advanced quantifications in particular regions) and morphological measurements (size, shape, etc.) (details in section 4). The great majority of studies rely on software or lab-developed scripts for image analysis maybe due to the quicker accessibility of results by automation and the accessibility to reproducible results. The remaining 4.21% realized only image observations or manual analysis such as cell counting with 1.05%. One can argue that observation does not allow quantification but contrary to manual counting, it is far less time consuming.

**Table 2:** Methodology to study cerebral organoids.

	**Microscopy**	**RNAseq**	**RTqPCR**	**Western blot**	**ELISA**
percentages	95%	50.5%	34.1%	26.2%	7.2%
advantages	visualize proteins	full transcriptome	studied gene	detect/identify proteins	detect antigens
inconvenient	sliced in 2D	only on thousand cells located	localization lost	localization lost	localization lost

## 3. Preparation and imaging

### 3.1. Sample Preparation

#### 3.1.1. Immunohistochemistry

Using a microscope may require the preparation of the 3D brain culture through fixation, slicing, and immunolabeling.

##### 3.1.1.1. Fixation

The fixation step allows the preservation and the long-term storage of tissues by stopping enzymatic reactions (Stanly et al., 2016). In our search, paraformaldehyde was the most commonly used fixation method for cerebral organoids.

##### 3.1.1.2. Slicing

Most of the protocols generating cerebral organoids and spheroids cut the samples in slices to facilitate imaging. In the 214 articles analyzed for this review, slices are cut between 5 and 50 μm. Slices are realized with different apparatus depending of culture conservation method: cryostat or microtome for frozen samples in the major cases (Mansour et al., 2018); microdissection laser microscopes when only a region is used (Buchsbaum et al., 2020); and a few use vibratome for cultures stored in PBS and agarose (Monzel et al., 2017; Berger et al., 2018; Gomez-Giro et al., 2019; Logan et al., 2020; Nickels et al., 2020; Smits and Schwamborn, 2020). Paraffin-embedded methods are rarely used on cerebral organoids due to the size of these cultures (less than a few millimeters).

In order to avoid slicing and to image a full cerebral organoid in a single acquisition, Durens et al. (2020) created a protocol aiming at reducing the organoid thickness to around 100 μm. This protocol enables imaging by a single acquisition with high-throughput imaging systems, such as confocal microscopes.

##### 3.1.1.3. Immunolabeling

Immunolabeling is a crucial biochemical step to prepare samples for the detection and the localization of an antigen—often a protein—inside a cell, a tissue, or an organ. To detect these antigens, a complex of antibodies targeting them are tagged. Fluorescent tags are used for confocal microscopy but an enzyme that catalyzes a colored reaction can be used for other microscopic methods, less used to study 3D brain cultures.

Immunolabeling is used in 3D brain cultures to detect a cell components such as nuclei (Gomez-Giro et al., 2019), microtubules (Buchsbaum et al., 2020), or mitochondria (Daviaud et al., 2018); a given cell type (neurons Smits et al., 2019 dopaminergic ones Bolognin et al., 2019, microglia Ormel et al., 2018, oligodendrocytes Marton et al., 2019, astrocytes Watanabe et al., 2017); or an extracellular marker (Lin et al., 2018b). Regions are also identified thanks to immunolabeling, with the combination of different cells markers (Li et al., 2017a; Anastasaki et al., 2020). Marked cells allow to monitor the tumor invasion inside cerebral cultures (Liu et al., 2020).

#### 3.1.2. Clearing of Organoids

To study a whole 3D sample without cutting, an old practice from the early 1900s consists of rendering it transparent: this method is called clarification. There are 4 main clarification protocols: based on organic solvents (OS), high-refractive index aqueous solutions (HIAS), hyperhydrating solutions (HS), and tissue transformation (TT). To find out more about each of the cited protocols, you can find more information in Matryba et al. (2019). Clarification is not commonly used for cerebral organoids: only 4% of articles use it (Table 3).

**Table 3:** References of articles using clarification on 3D brain cultures and corresponding image analysis between January 2018 and June 2020.

**Reference**	**Clarification category**	**Image analysis**
Sloan et al. (2018)	OS	cell migration
Masselink et al. (2019)	HIAS	fluorescence intensity and regional marker observation
Rakotoson et al. (2019)	HIAS or HS	nuclear detection and intensity
Sakaguchi et al. (2019)	TT	observation of markers
Krieger et al. (2020)	Hybrid HIAS and HS	tumor invasion
Buchsbaum et al. (2020)	OS	cell migration
Wilpert et al. (2020)	HIAS	observation of marker intensity

*Protocol abbreviations are as follows: HS, hyperhydrating solutions, TT, tissue transformation, HIAS, high-refractive index aqueous solutions, OS, organic solvent*.

The major drawback of this method is the time required by the protocols; the transparency varies over time and is tissue dependent; protocols can modify the morphological aspect of the culture, inducing over-sizing or shrinking; and some reagents are not compatible with the use of some immunolabelings. Nevertheless, clarification protocols are widely developed for the study of other organs models and even tumoral spheroids (Schmitz et al., 2017; Boutin et al., 2018; Costa et al., 2019; Nurnberg et al., 2020).

Clarified 3D brain cultures are acquired with confocal (mono-photon), multiphoton, or light-sheet microscopy.

### 3.2. Imaging Techniques

High-quality images are necessary to perform reliable analyses on 3D brain cultures. Bright-field, confocal, and light-sheet microscopy are the most often used modalities in this context (Table 4). We do not further describe microscopic methods not reaching 2% of use, such as inverted and phase contrast microscopy; those are grouped in the "others" category. The microscope used to acquire an images is chosen based on brain culture type, more specifically the thickness and preparation (Thorn, 2016), as well as the desired analysis to be performed.

**Table 4:** Percentage of articles per microscopy and per task performed for the analysis of cerebral organoids.

**Task**	**Bright-field**	**Confocal**	**Light-sheet**	**Not mentioned**	**Other/None**	**Total**
Observation	0.84	3.79	0.42	0.42	4.1	9.47
Morphology	3.79	19.16	2.11	2.32	9.4	36.84
Quantification	0.42	30.53	0.00	4.21	12.5	47.58
None	–	–	–	–	–	6.1
Total	5	53.5	2.5	6.9	32.1	100

#### 3.2.1. Bright-Field Microscopy

Bright-field microscopy is used to observe shape (Monzel et al., 2017) and surface parameters (Iefremova et al., 2017) of 3D brain cultures. On other 3D organ cultures, these images are also used to measure the overall size with automatic methods (Borten et al., 2018; Kassis et al., 2019; Hasnain et al., 2020). In such cases, samples do not require any particular preparation. Cultures can be examined without staining and the illumination does not alter the true colors of the sample. This system is simple and practical to use.

The light source is emitted below the sample and contrasts are created by the absorption of light in the sample. The in-plane resolution does not exceed 2 μm.

The issue often met using Bright-field microscopy is its 2-dimensional nature: although very useful for length and areas measures, only partial shape measures can be realized as the 3-dimensional information is not captured. Another problem is that the quality of the observation is reduced when the contrast is too high, creating distortions in the image. At low contrast, most of the cells are not observable as they are not stained. Confocal microscopy, for example, is better suited for cell observation.

#### 3.2.2. Confocal Microscopy

The most commonly used fluorescence microscope for 3D brain cultures is the confocal microscope (Table 4). The acquired images are analyzed to measure various parameters at the sub-cellular level such as intensity (Raja et al., 2016), shape (Cullen et al., 2019), surface (Karzbrun et al., 2018), cell distribution (Qian et al., 2016), or for 3D reconstruction (Monzel et al., 2017). Confocal microscopy allows the study of samples in the third dimension, which is impossible in bright-field. This optical microscope acquires images at low depth of field (around 500 nm). A laser sweeps the objective via a reflecting mirror. The beam goes through the sample to be imaged and a diaphragm reduces the light received by the sensor to the desired field of view. The whole image is acquired as a mosaic, making possible leveling down the sample plate of an increment of *z* to image the depth of the culture, and sweep another image. As a result, these stacked images can be used to reconstruct the 3D volume, enabling measures of 3D parameters characterizing culture structural properties. Immunolabeling via fluorescent tags is necessary to observe confocal images, contrary to bright-field, which conserves the natural color of samples.

One of the principal issues of confocal microscopy is the long acquisition time, particularly for in-depth imaging (in the *z* plane) where several hours per slice can be necessary. Moreover, only the first few slices produce a sharp signal. For these reasons, some teams prefer to use light-sheet microscopy for 3D culture imaging even though it requires a longer and more complex sample preparation protocol.

#### 3.2.3. Light-Sheet Microscopy

Light-sheet is commonly used to observe 3D samples. However, only 3% of cerebral organoid studies rely on this imaging method, mainly because of the high cost of the device and samples preparation. The illuminating laser source is in the acquisition plane, forming a light-sheet between 4 μm and 10 μm of depth, and of the sample width. The light-sheet is divided in 3 sub-beams (to limit artifacts) that converge toward the sample.

Light-sheet microscopy can acquire organoid images but the in-plane resolution and the light depth penetration are not sufficient to reconstruct a connectivity map according to Poli et al. (2019). For spheroids, which are 4 times smaller than cerebral organoids, the imaging of clarified data is feasible by light-sheet or confocal microscopy (Boutin et al., 2018; Costa et al., 2019).

#### 3.2.4. Other Imaging Methods

Others methods are sometimes used to study cerebral organoids for live imaging (Lancaster et al., 2013), to acquire Ca++ activity (Sakaguchi et al., 2019), or to monitor permeability to certain molecules (Bergmann et al., 2018).

## 4. Image Analysis

The aim of cerebral organoids image analysis is to quantify and characterize cell types (stem or proliferative cells, neuronal populations, oligodendrocytes, astrocytes, microglia or epitheliums), cells components (nucleus, neurites as dendrites or axons, mitochondria, synapses), pathological markers of specific disease, cell migration, permeability of tissues to specific molecules, necrosis, and structure formations inside the core of culture. In case of group studies, analysis is used to compare size, shape, and dimensions between cerebral organoid groups. In some cases, these results are used to complete and validate information obtained with another method (RTqPCR, ELISA, etc).

**Figure 5:**
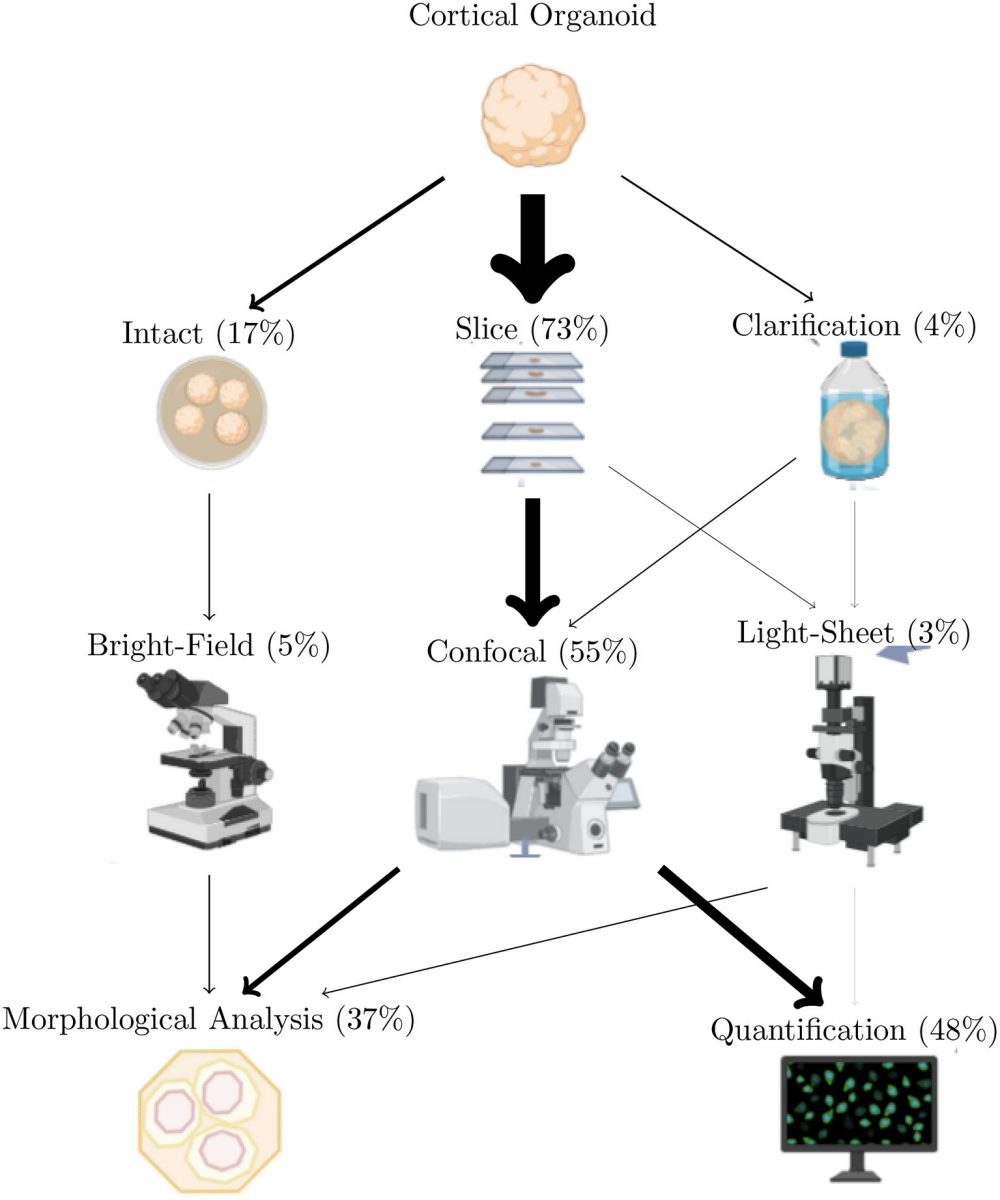
Imaging techniques most used to study cerebral organoids. Arrows width is proportional to use of the methods over the reviewed articles. Totals are less than 100% since only the most used methods are included in this figure. Also, a given article can describe multiple analyze types.

Pre-analysis stages are sometimes required to prepare data for future investigations. For example, 3D-reconstruction from acquired slices avoids counting cells multiple times when they appear in multiple *z* planes (Kartasalo et al., 2018). 3D-reconstruction also allows the visualization of the multi-view images acquired from light-sheet microscopy (Dobosz et al., 2014). Reconstruction methods from histological slices are based on different features: Fourier, blob, or high level features. Validation methods are based on observation, landmark detection, or measures of overlaps (Pichat et al., 2018). After pre-processing, cerebral organoid images are processed with different methods described in this section. As previously mentioned, the two main tasks performed on these images are quantification and morphology (Figure 5).

### 4.1. Quantification

Quantification is the main analysis realized on cerebral organoid images (occurring in more than 47% of the reviewed studies, see Table 5). Quantification includes markers detection and identification of counting, calculation of intensity, and advanced methods for studying cerebral organoid regions.

**Table 5:** Quantifications performed on cerebral organoid images, given in percentage of the reviewed articles.

**Quantification**	**Type**	**Percent**
Counting	Cell	20.84
	Protein	8.21
	Nuclei	6.74
	Synapses	1.89
	Pathological	1.05
	Mitochondria	0.21
Density	Various markers	7.16
Total		47.58

#### 4.1.1. Counting

Counting is performed on specific cells or cell components. In this section, after describing the different quantified structures, we detail some of the counting methods described in the literature.

##### 4.1.1.1. Biological Structures

- Neurons and glial cells: Cell counting constitutes 20% of image analysis performed on cerebral organoids (Table 5). Brain growth can be tracked by counting markers of neural stem cells (Smits et al., 2019), proliferative cells (Cullen et al., 2019), or differentiated neurons (Berger et al., 2018). In addition to neurons, the brain is constituted of glial cells. Astrocytes are responsible for nutrition and neuronal communication while oligodendrocytes constitute the neuronal myelin gain. Both cell types have been quantified in previous studies (Cullen et al., 2019; Kim et al., 2019; Nickels et al., 2020; Zhong et al., 2020). Counting of microglia—another kind of glial cell responsible for immunity—has also been investigated (Brownjohn et al., 2018; Ormel et al., 2018). Quantifying organoids microglial cells can help study both their development and their interaction with neurons in case of disease. The last kind of glial cell, constituting the epithelium barrier of brain cavities, is also quantified in choroid plexus organoid models (Pellegrini et al., 2020). Their function of secretion is measured in this previous article by quantifying a typical molecule of transport (transthyretin) only expressed in choroid plexus.
- Nuclei: The nuclear compartment present in eukaryotic cells contains its genetic information. Brain culture development is assessed by counting the total nuclei number (and therefore the total cell number) in a slice, a particular region, or an entire brain culture (Bagley et al., 2017; Berger et al., 2018; Park et al., 2018; Jacob et al., 2020; Kielkowski et al., 2020; Qian et al., 2020). Identifying nuclei also allows identifying the proportion of apoptosis (cell death), helpful to quantify organoid viability (Smits et al., 2019; Nickels et al., 2020; Pedrosa et al., 2020; Zheng et al., 2020). A similar process with a counter-stain permits the characterization of the neuronal population density. For example, Smits et al. (2019) and Berger et al. (2018) segment nuclei and dopaminergic neuronal markers in midbrain organoids to determine the neurons proportion of their models.
- Synapses: Connective zones between neurites of neurons where the information is transmitted. Number of synapses and their functionalities are altered in case of organoid models of various diseases (Ghatak et al., 2019; Gomez-Giro et al., 2019).
- Pathological and physiological proteins: Proteins constitute cells and play various roles in transmitting information or regulating factors. In cerebral organoids, proteins are quantified to identify a particular cell component such as regulating factors of transcription or tubulin markers (Lancaster et al., 2013). To quantify diseases markers, a key is to count any excessive or insufficient amount of physiological marker, or identifying a pathological marker. For example, the number of A*beta* puncta is used to identify Alzheimer markers in cerebral organoids (Lin et al., 2018b).
- Mitochondria: These are involved in energy conversion resulting from cellular respiration. Mitochondrial abnormalities caused by genetic mutations in some diseases like in Parkinson organoid models (midbrain organoids) can result in cell death (Bolognin et al., 2019).

##### 4.1.1.2. Methods

Counting cell markers relies on many different procedures. For example, different studies use the following steps: first, images are denoised using median filtering. Second, a Gaussian filter is applied in order to obtain a mask for the marker. Then a median filtering is used on masks, and connectivity is searched to remove small connected components (Berger et al., 2018; Bolognin et al., 2019; Smits et al., 2019; Nickels et al., 2020). Finally, expression levels of markers are expressed in pixels or percentage, and sometimes are normalized by the expression level of nuclear markers.

Another way to count cells consists of binarizing each channel using Otsu thresholding (Otsu, 1979), and separating overlay cells using watershed (Meyer, 1994). Images are then denoised and channels are overlayed to count cells and calculate ratios (Cullen et al., 2019).

Most nuclei identification methods use a foreground and background image, which are first convolved with a Gaussian filter, then substracted from one another to obtain segmented nuclei (Berger et al., 2018; Bolognin et al., 2019; Nickels et al., 2020). In some cases, the Gaussian filtering is applied directly on the Hoechst channel (Smits et al., 2019).

A way to quantify synapses is to manually segment them using a specific software (Quadrato et al., 2017; Gomez-Giro et al., 2019). Others choose to co-localize pre-synaptic and post-synaptic punta inside a population of neuronal cells by semi-automatic tools and quantify them per micrometer of neurite length (Ghatak et al., 2019).

In order to quantify mitochondria, Bolognin et al. (2019) segmented the plate of organoid culture, cell nuclei, cell, and then a mitochondrial mask was defined via a difference of Gaussians. Masks were refined using a sequence of operations (connected component removal, erosion, and skeletonization).

#### 4.1.2. Intensity

In order to quantify the proportion of cell components or molecules inside brain cultures, marker intensity measure has been proposed (around 7% of the image analysis). Different markers can then be measured: neurotransmitters (Sartore et al., 2017; Jorfi et al., 2018), molecule transporters (Wilpert et al., 2020), infiltration of tumors (Liu et al., 2020), nuclei (Rakotoson et al., 2019), or pathological markers (Lin et al., 2018b).

To measure the neurotransmitter intensity, the mean gray value of this specific marker is measured in three points of each cerebral organoid border, delimited by a rectangular selection. This fluorescence intensity is then normalized for the tissue background (Jorfi et al., 2018). To assess the neurotransmitter intensity per particular neurons, this parameter is normalized to total neuronal intensity (Ghatak et al., 2019). To quantify the tumoral infiltration regions, the fluorescence intensity is thresholded (Liu et al., 2020). For intensity of nuclear markers, background image was subtracted from stained one, the image (originally in 16 bits) is converted in 8-bit gray-scale, and the intensity of this marker is measured (Stachowiak et al., 2017).

#### 4.1.3. Advanced Regional Quantification

When Lancaster et al. (2013) generated the first cerebral organoid, they discovered the presence of various brain regions, similar to the ones already described in human brain. It is possible to identify regions using a combination of different markers, marker density, or marker location. Pasca et al. (2015) were the first to quantify different types of cells inside cortical spheroid regions: a ventricular zone (VZ), a deep layer, and a superficial layer. One year later, Raja et al. (2016) counted nuclei expressing a caspase to determine the cell death from the center to the external cortex of a cerebral organoid. Indeed, markers of cell death and proliferation are often measured in VZ and SVZ regions (Anastasaki et al., 2020; Jacob et al., 2020; Qian et al., 2020; Zhang et al., 2020). Other articles also calculate the percentage of particular neurons in VZ, SVZ, outer SVZ (Li et al., 2017a) or CP (Zhang et al., 2019). With the emergence of fused specific region organoid, Bagley et al. (2017) expressed the percentage of various fluorescent markers in dorsal and ventral forebrain organoids.

As of today, regional quantification mostly remains on a semi-automatic process (Albanese et al., 2020). All of the articles cited use imageJ after a manual extraction of the region of interest. Regional organization is also scored manually by three authors in "no organization," "geographic segregation," and "laminar structures" to determine the degree of differentiation (Cullen et al., 2019).

Between January 2018 and June 2020, we only found classic segmentation methods to identify cell components. It would be interesting to test various segmentation methods to identify the most adapted to accurately identify cellular components.

### 4.2. Morphological Analysis

**Figure 6:**
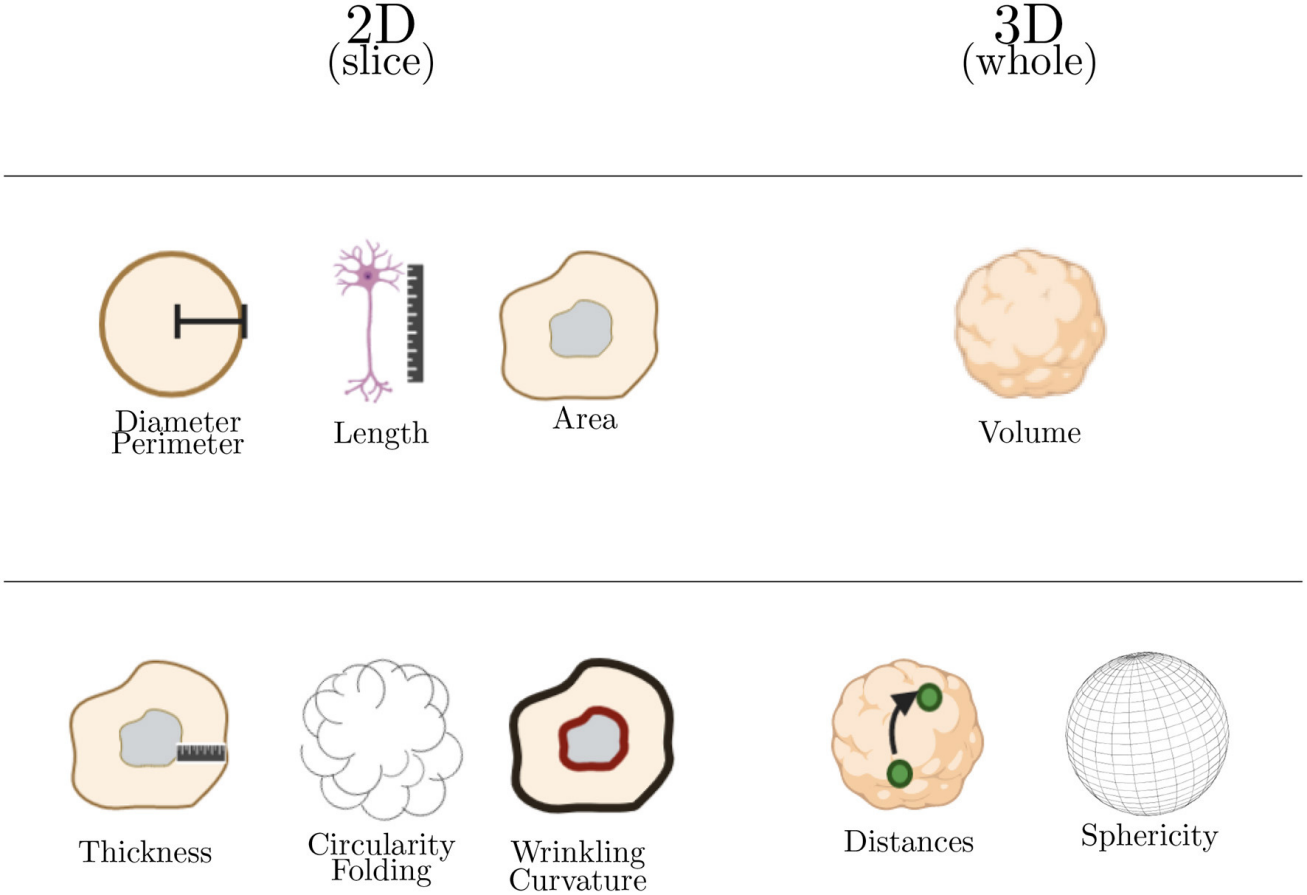
Summary of major morphological analysis performed on cerebral organoids. The first line in this table corresponds to basic morphological analysis and the second one to advanced morphological analysis. Basic parameters are used to calculate the advanced ones.

Morphological analysis represent approximately 37% of the studies of 3D cerebral organoids images and are summarized in Table 6. Upon these morphological parameters harvested, two categories are further detailed in this section: basic and advanced metrics containing 2D (diameter, perimeter, length, area, folding, wrinkling, curvature, and circularity) and 3D analysis (volume, sphericity, and distances) (see Figure 6).

**Table 6:** Morphological analysis performed on cerebral organoid images, given in percentage of the reviewed articles.

**Type**	**Analysis**	**Percent**	**Dimension**
Basic	Diameter	4.84	2D
	Perimeter	0.84	2D
	Unspecified size	2.95	2D
	Distances	4.21	Mix
	Neurite	2.95	2D
	Radialization	0.42	2D
	Ventricles	1.68	2D
	Nuclear Morphology	0.21	2D
	Area	11.58	2D
	Volume	1.26	3D
Advanced	Thickness	4.63	2D
	Folding	0.63	2D
	Tortuosity	0.21	2D
	Curvature and Wrinkling	0.21	2D
	Circularity	1.05	2D
	Sphericity	0.42	3D
Total		36.84	

#### 4.2.1. Basic Metrics: Two-Dimensional Analysis

Some studies investigated organoid global growth by measuring size indices to identify the state of growth and well being of the culture, as well as to compare methods of culturing or disease models of cerebral organoids.

##### 4.2.1.1. Diameter and Perimeter

Diameter and perimeter are measured in (6%) of cerebral organoid articles. They are part of tools to measure the size of cerebral organoid to evaluate their growth or to compare different groups of culture (healthy and disease models, for example). Indeed, their size is evaluated by their diameter (Monzel et al., 2017; Sartore et al., 2017; Sivitilli et al., 2020) or perimeter (Buchsbaum et al., 2020) on bright-field images. Others authors use confocal microscopy to measure the size in term of perimeter (Iefremova et al., 2017).

To measure these parameters, semi-automatic tools are provided in some software. One of the method is to sample diameter twice in a perpendicular angle using the line tool of FIJI, on maximum *z*-projections made from image stacks acquired by confocal microscopy (Schindelin et al., 2012). In bright-field microscopy, perimeter of an element inside an image is measured on boundaries of manual or semi-automated selected regions. For diameter, the longest distance between two points of a selected region is measured. These measures had to remain in early stage of development due to heterogeneous shape in later stage in this culturing model.

Such metrics could become an indicator of cerebral organoid shape only in early stages. Nevertheless, in other kind of organoid models, diameters are an indicator of their shape all along their growth as most of them stay spherical and a few are elliptical (Kassis et al., 2019). In this article, intestinal organoids are identified and their diameters are measured thanks to deep neural network based on anchor boxes and features pyramidal network from Lin et al. (2018a). Some software are developed solely to measure the spheroid perimeter (Chen et al., 2014).

##### 4.2.1.2. Length

The first form of measuring distances is measuring it at cell scale. Measuring cell component allows to identify characteristic of growth culture. Researchers measure, for example, neurites (Cullen et al., 2019; Xiang et al., 2019; Ao et al., 2020; Durens et al., 2020) or cilium length (Zhang et al., 2019). Using lengths, ratio can be calculated to compare neurites in different regions inside cerebral organoids (Xiang et al., 2019), or to evaluate the direction of growing of this cell component (Durens et al., 2020).

To measure the length of cell components, semi-automatic tools are used to define the boundaries of each of neurites or cilium, and distances between the two boundaries are calculated.

##### 4.2.1.3. Area

The surface area better represents cerebral organoids shape in latter stages than other 2D parameters, being more acute on non-spherical shape. Area is the most calculated morphological parameter on 3D brain culturing (12%), and is used to compare various cerebral organoids growth in different conditions or groups (Watanabe et al., 2017). At the sample level, the surface area of 3D reconstruction of light-sheet microscopic images can be performed (Li et al., 2017b; Wang et al., 2020b). Nevertheless, some authors prefer measuring regions (VZ and SVZ and CP) in term of areas on slices to follow their growth (Iefremova et al., 2017; Watanabe et al., 2017). Particularly ventricular lumen area is measured in order to know the state of 3D brain culture or to test a pharmacological component (Qian et al., 2016; Iefremova et al., 2017; Sartore et al., 2017; Di Matteo et al., 2020). Area can also serve to determine the culture viability. The necrotic and viable areas are measured in the case of comparisons of two kind of culturing Berger et al. (2018). The calculation of an expressed marker area without considering the regional segregation can also be done (in entire slices). The area of a kind of neurons or glial cells (Park et al., 2018) and the area of all the nucleus (Cullen et al., 2019) are another example of this kind of measurement. Of note, measuring area enables the evaluation of co-localization of some markers (Ao et al., 2020) like presynaptic and post-synaptic ones.

To identify the growth of cerebral organoid culture, authors calculate the total surface area of the whole organoid. Regions of interest are surrounded manually around the entire organoid from a bright-field microscopy image, and thanks to an imageJ module ("Area Measurements of a Complex Object"), the surface area is calculated (in pixels) (Gomez-Giro et al., 2019; Zhang et al., 2020). Viability of cultures can be assessed both at regional or cellular level. Berger et al. (2018) choose a typical fluorescent marker not expressed in necrotic core region and measure its surface expression related to the total surface area. This parameter is measured as the minimum area in pixels that an object must have after its selection, thanks to semi-automatic tools (Zen software). For cell viability, areas of some cell component markers (such as plasmic membrane or enzyme) are also calculated. To that aim, Cullen et al. (2019) convert the two channel corresponding to plasmic membrane and enzyme in 8 bits images, then binarize images to obtain cell shape regions. The area of these two markers is then quantified, and their ratio is calculated. Synapses quantification can be achieved using marker areas co-localization. Synapse areas are, for example, calculated by overlapping Homer (post-synaptic) and Bassoon (pre-synaptic) channel signal in the case of assembloid of organoids using a lab-developed tool (Sloan et al., 2017).

For particular cerebral organoids, areas are even calculated. For example, in fused ventral and dorsal forebrain organoids, areas of typical expressed markers are also calculated (Bagley et al., 2017). For the blood–brain barrier organoids, areas are equally measured, particularly the core area by measuring it at 50 μm from the surface. A scale bar is used as a reference to correctly assess the distance (Bergmann et al., 2018). In mammalian, colon and intestinal organoids, the whole area of the entire organoid digitized after bright-field imaging is calculated (Borten et al., 2018; Ren et al., 2018; Hasnain et al., 2020). As an example, for Borten et al. (2018), after a segmentation of colon organoids (by a conversion, opening-closure, thresholding, filtering to denoise, filling holes, denoising, and removing debris), the surface area of identified region of interest is measured.

#### 4.2.2. Basic Metrics: Three-Dimensional Analysis

Measuring the cerebral organoid size in 3D is also possible in light-sheet images, where the volume of this 3D brain culture is assessed (Li et al., 2017b; Wang et al., 2020b). Only few authors calculated this parameter, possibly because this imaging modality is poorly used. Indeed, 2.5% of articles use light-sheet, and 1.3% calculate the volume of cerebral organoids (Table 6).

The outline of the cerebral organoid is delineated and used to compute both volume and surface area, with semi-automatic tools (Li et al., 2017b). Such metric could be use to indicate if an antitumoral treatment works like it was made for spheroids. However, the number of spheroids is too important to semi-automatically or manually measure volume when performing drug testing. Kalaydina et al. (2019); Wojaczek et al. (2019) use deep learning method based on the YOLOv2 architecture (using anchor boxes instead of fully connected layers) (Redmon and Farhadi, 2016) to identify spheroids and calculate their volume *V* from the radius *r*, assuming a perfect sphere. Manual calculation of the radius *r* was made by measuring the diameter twice for each spheroid, then averaged and divided to obtain *r*, using a scale bar as a reference. After automated identification, coordinates of predicted bounding box enable the measurement of radius and the volume calculation (Kalaydina et al., 2019).

#### 4.2.3. Advanced Metrics

##### 4.2.3.1. Length Distances and Thickness

Advanced specific distances are calculated in Cederquist et al. (2019) to identify the cerebral organoid patterning. First, the center of mass (CM) of a factor-organizing cells is computed, inside a grid applied on the image. The CM is a function of its mean gray value intensity and the total intensity. After choosing a marker of a typical protein, intensity is thresholded and regions of interest (ROIs) are identified. Finally, the Euclidean distance between each ROI and the CM is obtained.

The second kind of distance is the neuroepithelium thickness. In cerebral organoids, this thickness is used to characterize an organoid model (Watanabe et al., 2017; Sakaguchi et al., 2019; Buchsbaum et al., 2020; Di Matteo et al., 2020; Zhang et al., 2020) and to follow the patterning of the culture (Cederquist et al., 2019) or the effect of various culturing on the growth of the regions contained in it (Qian et al., 2020).

A specific feature of the neuroepithelium thickness is the relative thickness *R*_*thick*_, which is the ratio of the total layer thickness *TL*_*thick*_ over the VZ region thickness *VZ*_*thick*_ (Zhang et al., 2019):

(1) $$\begin{array}{r} R_{thick}=\frac{TL_{thick}-VZ_{thick}}{TL_{thick}} \end{array}$$

Another way to calculate the relative VZ thickness is defined as the ratio of VZ thickness to VZ plus outer layer thickness (Qian et al., 2016).

##### 4.2.3.2. Circularity and Folding

The shape of the cerebral organoid is one of the parameter used to distinguish it from spheroids, and a marker of later stage of the cerebral organoid growth. Circularity (*C*) is a shape parameter measured in the early stage (day 6) of development in intact cerebral organoids, and is defined by Yoon et al. (2019) as:

(2) $$\begin{array}{r} C=4\pi \cdot {\frac{A}{P}}^{2} \end{array}$$

where *A* is the object area and *P* is the perimeter. An index of 1 reflects a perfect circle.

Human cortical surface is characterized by folding (gyri and sulci), which is not always present in mammalian models (Kelava and Lancaster, 2016a). To determine if a cerebral organoid model reproduces gyrification, Li et al. (2020); Wang et al. (2020b) quantify folding. On bright-field or in higher magnification view images, the Canny edge detector is used to extract edges. Once edges are found, their total length is used to compute a folding index (Wojaczek et al., 2019).

##### 4.2.3.3. Wrinkling and Curvature

Wrinkling occurs at two brain formation stages: during the emergence of folds along the neural tube, and during the expansion of surface area. Measuring wrinkling is a relevant index to characterize diseases such as lissencephaly. Karzbrun et al. (2018) calculate the wrinkling and the curvature inside cerebral organoid. 2D wrinkling is the measure of the real perimeter of the organoid divided by the total maximal perimeter as a circle containing the organoid. The curvature is defined as the average of the tangent angle *θ*(*r*) derivative along the surface of inner and outer neuroepithelium perimeter contour *ηrθ*(*r*).

##### 4.2.3.4. 3D: Sphericity and Distances

For 3D images, circularity cannot be characterized, hence the identification of brain gyrification uses the sphericity (how spherical an object is) on light-sheet images (Li et al., 2020; Wang et al., 2020b). The calculation of sphericity, *ϕ* originally generated by Wadell in 1932, is defined as the ratio of the cell surface area of a sphere over the cell surface area of a particle, with *V* the volume of the particle and *A* the surface area of the particle:

(3) $$\phi =\;\frac{\pi^{1/3}{\left(6V\right)}^{2/3}}{A}$$

The latest measure performed on cerebral organoids evaluates the tumor propagation in some models. The distances between tumoral cells or between them and the center of the cerebral organoid is computed. From binarized images, several steps are then performed: exclusion of single cells (using by connected components), holes filling, organoid surface approximation (by a Delauney triangulation). Normal distances between tumoral cell voxels is then calculated (Krieger et al., 2020).

#### 4.2.4. Summary on Morphological Parameter Extraction

Over the considered time range (January 2018 to June 2020), we only found methods focusing on classic extraction of shapes. More recently (Albanese et al., December 2020), authors extracted ventricular region of cleared organoids using a deep-learning approach (U-net architecture Ronneberger et al., 2015). This original work paves the way to deep-based approaches and clearly shows the potential of such methods. Similar methods could potentially be used for all types of cerebral organoids structures.

## 5. Software

### 5.1. Pre-Analysis Software

Most imaging platforms include a software able to perform pre-analysis. For example, the tiles module and the position module of the Zen software can be used to reconstruct multi-view images in 3D (Watanabe et al., 2017), while the NIS imaging software (Nikon) can measure the size of cerebral organoids (Berger et al., 2018).

However, these software packages are generally not adapted to perform the tasks variability required by researchers who want to analyze cerebral organoid imagings. To analyze images, neuroscientists choose dedicated software depending on the study topic, imaging type, ease of use, source code flexibility, their computing knowledge, and budgets. ImageJ, Matlab, CellProfiler, and Imaris are the most used software solutions in this context, as shown in Table 7.

**Table 7:** Software used to analyze cerebral organoid images.

**Software**		**Open source**	**Automatism**	**percent**
imageJ / Fiji		yes	semi-automatic	51.37
Matlab		no	automatic	4.42
	CellProfiler	yes	semi-automatic	0.84
	Vast	yes	semi-automatic	0.42
Imaris		no	semi-automatic	3.16
Visiopharm		no	automatic	≤ 0.2
ImageScope		yes	automatic	≤ 0.2

### 5.2. ImageJ/Fiji

ImageJ is an open-source software, which can run on all the main operating systems (Windows, macOS, Linux/Unix). It does not require knowledge in coding and the interface is somewhat user friendly; for example, it supports "drag and drop" of the image to analyze. ImageJ is the most widely used software for the analysis of 3D brain cultures (used in over half the articles surveyed, see Table 7). The most popular modules include the "cell counter" plugin, the "particle" counter, the "length" and "area" measurement functions, the "ROI tool," and the "Canny edge detection" to measure folding density.

For those who need further analysis, the advantage of this software is the possibility to code macros in Java to automate analysis or to create new tools (Raja et al., 2016; Ormel et al., 2018). One drawback is that some file extensions require additional plugins to be handled (for example, bioformat files) while in-house extensions are not handled at all. Also, ImageJ performances are impacted when used with large images and may require increasing memory allocation.

Theoretically, it is possible to perform 3D analysis with the "ImageJ3Dviewer" plugin. However, to our knowledge, these tools have not been used for the analysis of 3D brain cultures.

### 5.3. Matlab

Matlab is a numerical computing environment and proprietary programming language widely used by the scientific community, for example for image and data processing or simulations ^1^. Matlab can also run on the main operating systems. Many toolboxes exist and can be used to develop new tools. Matlab is more versatile and faster than the other software on large amounts of data, but it requires specialized knowledge to develop and validate new tools. Matlab is the second most used software (with 5% of use) and has been used for a wide range analysis tasks: nuclei segmentation (Smits et al., 2019); cell segmentation (Bolognin et al., 2019); puncta co-localization (Sloan et al., 2017); curvature, folding, and surface measurement (Karzbrun et al., 2018); and tumoral cell dispersion evaluation (Krieger et al., 2020).

#### 5.3.1. CellProfiler

CellProfiler is an open source software developed in Matlab; it thus requires a Matlab license. Many plugins are available and used by different teams analyzing 3D brain cultures (Park et al., 2018; Pedrosa et al., 2020). The major inconvenient is that not all image formats are currently accepted. Specific scripts must be developed, but new plugins can be coded in Matlab as mentioned before.

#### 5.3.2. Vast

Vast ^2^ is a Matlab-based semi-automatic segmentation tool for 2D and 3D images and is used to segment images from transmission electron microscopy, including segmentation of synaptic compartment in cerebral organoids (Quadrato et al., 2017).

### 5.4. Imaris

Imaris is a commercial software that allows 3D and 4D (along the time) analysis of cell cultures, but it remains a semi-automatic tool. User selects objects inside images to detect and process them. Imaris is used in 3% of the articles surveyed for this review, and is particularly used to reconstruct images in 3D (Kadoshima et al., 2013; Renner et al., 2017), to count cells (Li et al., 2017a), and to quantify volumes, surface area, and sphericity (Li et al., 2017b).

### 5.5. Other Solutions

Visiopharm^3^ is a commercial solution composed of a range of AI-based image analysis and tissue mining tools. It has been used on fluorescent cerebral organoid images to count cells (Stachowiak et al., 2017). ImageScope^4^ is a commercial automatic quantitative software for widefield microscopy, which is used to count pixels labeled with a specific marker for prion in a Creutzfield-jacob model of cerebral organoid images (Groveman et al., 2019).

Others methods have been validated for the study of non-cerebral organoids: Cytocensus for retinal organoids (Hailstone et al., 2020); OrgDyn for widefield images of mammalian organoids (Hasnain et al., 2020); OrganoSeg for 3D bright-field images of colon organoids (Borten et al., 2018). Most of these tools are based on image filtering and segmentation. Notably, OrgaQuant locates and quantifies the size distribution of human intestinal organoids in bright-field images based on a deep learning network (Kassis et al., 2019). Only recently a software was created to characterize the cytoarchitectures of cerebral organoids imaged by light-sheet microscopy (Albanese et al., 2020).

## 6. Discussion

This section gives an overview of the current limits in cerebral organoids generation, existing imaging solutions, and analysis methods and tools. We also present expectations for new image and volume analysis tools. Indeed, one of the key point in the context of image analysis of cerebral organoids is the feasibility of the analysis supported by the quality of generated images and on their imaging.

### 6.1. Cerebral Organoid Generation Limitations

Some limitations remain in the generation of cerebral organoids. The main limitation is the necrosis occurring during the growth of cerebral organoids due to lack of nutrients and oxygenation. Slicing the organoid and optimizing the culture medium have reduced this necrosis (Berger et al., 2018; Qian et al., 2020). However, the lack of vasculature of cerebral organoids remains the root of the problem. In some protocols, cerebral organoids are transplanted in mice brains for irrigation (Mansour et al., 2018; Pham et al., 2018; Shi et al., 2020) while others generate blood–brain barrier organoids (Cho et al., 2017; Bergmann et al., 2018; Nzou et al., 2018) but these solutions lack the self-patterning of vessel generation. Recently, the theoretical elucidation of this problem has been exposed based on two models of gradient diffusion of the vascular endothelial growth factor (Hong and Do, 2019). A recent study also documents the generation of telencephalic and choroid plexus organoids allowing the production of cerebrospinal fluid (Pellegrini et al., 2020). A combination of these barriers in a cerebral organoid model could potentially increase its lifespan.

"Batch syndrome" and batch-to-batch variability as previously described are a major inconvenient for the commercialization and robust analysis of cerebral organoids. A prerequisite for commercialization consists of measuring size and morphological complexity (cf. 2.2) to validate the model (Choudhury et al., 2020). However, existing tools to measure the overall size present drawbacks like time consumption as they are manual or semi-automatic, making them unsuitable for mass production. Though the generation of this model is less than a decade old and not well stabilized, growth monitoring of cerebral organoids neglected for the benefit of articles comparing pathological and physiolocal cerebral organoid models. Almost half of the related articles and reviews included in this review are about pathological organoids (Figure 2 and Table 1). In others organ models, automatic tools have emerged to measure the size or to classify the morphology of others organ models (Borten et al., 2018; Kassis et al., 2019; Hasnain et al., 2020). We think a similar tool for cerebral organoids could help to measure and identify the growing step of cerebral organoids.

The large amount of cells to handle in generated cerebral organoids, in addition to their variability in numbers, also increases the difficulty in analyzing images (from 3,000 to 11,000 cells at 6 months depending on the protocol). Nevertheless, similar problems have already been addressed. For example, connectome has already been investigated for larger central nervous systems like drosophilae (25000 neurons and their projection), but also in part for the human brain (containing around 86 billion of neurons and their projections) (Maller, 2019; Scheffer et al., 2020; Rosen and Halgren, 2021). The Human Connectome Project requires to create collaborations between laboratories and a large storage capacity, as terabytes of storage are required in computing resources for a whole human brain. In order to investigate the development of cerebral organoid connectome under various protocols, we think it could be necessary to create a similar initiative collaboration, and biobanks dedicated to cerebral organoids images. Another review discussed about the benefit and limitations of conserving cerebral organoid generated or their cell contents inside biobanks (Li et al., 2020). It could help also to investigate, for instance, each neuropathological model created in cerebral organoids as it has been done for glioblastoma (brain tumor) cultures of patients (Jacob et al., 2020). Generation of cerebral organoids is not the only limitation of these models; to an image analysis point of view, the imaging remains an issue.

### 6.2. Preparation and Imaging Methods Limitations

#### 6.2.1. Preparation

Sectioning during preparation restricts the efficiency and throughput of organoids and spheroids (Pasca et al., 2015). The loss of bio-material is critical for these small cultures that do not exceed 4 mm in diameter for cerebral organoids and 0.5 mm for spheroids. Moreover, 3D reconstructions computed from these altered images can introduce a bias (Richardson and Lichtman, 2015). To avoid slicing and to obtain a full cerebral organoid image in a single acquisition, Durens et al. (2020) generate an organoid with a thickness of 100 μm. Another problem of classic immunohistochemistry methods is the poor diffusion of markers in the depth of cultures. A possible solution is to use clarification. Nowadays, only a few teams use this expensive solution on cerebral organoids (see Table 3). The aim in the near future is to use clarification in high-throughput platforms (Poli et al., 2019; Grenier et al., 2020). Very recently, out of the time scope of this review, cleared cerebral organoids were analyzed in one of this expected platform called SCOUT (Albanese et al., 2020). Authors also tried other clarified methods to analyze their cerebral organoids in 3D (Renner et al., 2020; Adhya et al., 2021). However, contrarily to the spheroids field, to our knowledge there is no article comparing existing clarification methods to find the most accurate one, allowing better image analysis on 3D brain cultures (Nurnberg et al., 2020). An appropriate clarification method applied on cerebral organoids could help to acquire images of quality and allow the most accurate 3D analysis.

#### 6.2.2. Imaging

Each of the various acquisition methods used on cerebral organoids has specific limitations. Bright-field microscopy is only used to analyze intact samples and is a powerful and simple acquisition modality to identify 2D morphology and follow the growth, however not suited for inner cells study. The resolution of confocal microscopy is satisfactory only for the superficial sweeps while only a halo of markers are visible in the deepest views (Smits et al., 2019). Accordingly, only cell counting in a single acquisition plane is possible (Qian et al., 2020). Light-sheet microscopy is only used by few teams (see Table 4). This method is an expensive solution that requires to be tested on cerebral organoids clarified by various protocols before obtaining good quality data. This imaging method has been used in the recently published articles on only one clearing method (Albanese et al., 2020; Adhya et al., 2021). A comparison of images resulting from various clarification protocols in light-sheet and confocal modality, not already provided to our knowledge, could be an important step to identify the best methodology for the observation and analysis of 3D cerebral organoids. Such image acquisition methods diversity yields additional complexity in the automated analysis tools standardization (Table 4).

### 6.3. Analysis

Some authors chose to develop their own algorithm rather than using already available software modules (Stachowiak et al., 2017; Berger et al., 2018; Bolognin et al., 2019; Cullen et al., 2019; Smits et al., 2019; Krieger et al., 2020). In addition to software imaging and updates high costs, these are usually not optimized for their specific imaging modalities. Also, commercialized software source code is not always available, to be modified to fit custom needs. With regard to clarified samples images, only a few software are allowing 3D-data analysis (see section 5). Noteworthy is the fact that most of the existing solutions remain semi-automatic. In the actual context of data expansion and increase of organoids models (Ashok et al., 2020; Choudhury et al., 2020), the development of fast and automated tools is mandatory.

Indeed, manual characterization of spheroids, smaller than cerebral organoids, is time consuming (Soetje et al., 2020). In contrast, automated processing based on computational neural network (CNN) can provide real-time measures (Kalaydina et al., 2019; Wojaczek et al., 2019; Anagnostidis et al., 2020). In other imaging disciplines such as MRI brain tumor detection, similar methods are already widely developed (Gordillo et al., 2013).

Aside from quantification speed optimization, another benefit of CNNs is that they are not subject to human error (except from the manual annotation process). Nowadays, CNNs are used to measure size parameters from 3D intestinal organ models, (Kassis et al., 2019) or to count cells in retinal organoids (Hailstone et al., 2020).

Despite the fact that is widely developed for others 3D cultures, to our knowledge, only one article included deep learning methods in order to segment ventricules of their cerebral organoids (Albanese et al., 2020). However, the comparison of machine learning methods applied to cerebral organoids would bring precise information on analysis precision and reproducibility. Nevertheless, the lack of shared images databases precludes such a comparison (Chakradhar, 2016).

### 6.4. Need of Analysis Tools

Automatic monitoring during cerebral organoids development, although essential for their commercialization (Chakradhar, 2016) and management of the increase culture amount (Figure 1), is still lacking.

In others organ models, i.e., mammary organoids (Hasnain et al., 2020), automated tools allowed the discovery of various groups of morphology. Such classification would be interesting to highlight in cerebral organoids.

Studying the morphology and measuring the size of a cerebral organoid in 2D images can help to compare groups inside a study (Iefremova et al., 2017; Monzel et al., 2017; Watanabe et al., 2017). However, the tools used to that aim are still semi-automatic or manual. A possible answer lies in the use of CNNs, which can help identifying and characterizing cerebral organoids in the culture (Kalaydina et al., 2019; Kassis et al., 2019; Wojaczek et al., 2019; Anagnostidis et al., 2020; Soetje et al., 2020). These tools completed with transcriptome analysis in various locations inside some cerebral organoids blindly selected in a batch, could help to automatically validate the growing step of a cerebral organoid, in a productivity chain. Such a tool used in research would improve the speed of organoid groups comparison. Additionally, automated size and growth measurement could be helpful in other 3D cultures (organ models or spheroids), less complex in term of morphology (Friedrich et al., 2009).

Another interesting feature of cerebral organoids is the presence of regions mimicking similar human brain regions, but at random location, with shape variability and in random numbers (Lancaster et al., 2013). Regional quantification has already been performed in two dimensions with semi-automatic tools (Anastasaki et al., 2020; Jacob et al., 2020; Qian et al., 2020; Zhang et al., 2020). Conversely, automatic 3D structures extraction has not been done yet, except for ventricular regions (Albanese et al., 2020). To observe or quantify molecules in specific brain regions, researchers use atlases on the assumption that structures localization and shapes are identical to the ones found in a healthy subject. Such assumptions are not valid for cerebral organoids, because of the previously mentioned variability. Moreover, atlas creation process is a complex task, even in the case of *in vivo* models or human brain (Johnson et al., 2010; Bazin et al., 2020; Wang et al., 2020a). Recently, some authors developed a brain atlas based on deep learning in order to automate the segmentation of mice brain regions, which are variable in size and shape (Iqbal et al., 2019). This study demonstrates the feasibility of localizing brain structures despite mild brain variability, and could be translated to cerebral organoid study.

Additionally, the minimal density of markers defining a region in 3D would be interesting to highlight. Such characterization could help identifying unknown functional and architectural markers, as mentioned in Grenier et al. (2020), with the perspective of generating a high-throughput deep learning-based image analysis platform for drug testing.

Such platform could benefit many other applications. Defining regions with a reduced number of markers on a single sample could leave room for another marker, more relevant for a specific study. Moreover, organoids structures are manually extracted to count markers, or are cut to analyze in RNAseq (Sloan et al., 2018; Buchsbaum et al., 2020). Nevertheless, structures are microscopic, and the tools enabling the selection of regions depend on the accuracy of the operator. This becomes particularly critical when regions are cut with laser microscopes. Precision in cerebral organoid cutting could be increased using automatic region identification, or error correction through a dedicated analysis tool.

Automated quantification of cells and their components would be of great interest, as such measures remain the main analysis realized on cerebral organoids (Figure 5). While cell counting is the principal quantification realized on cerebral organoids (cf. Table 5), authors only use classical segmentation (thresholding, watershed for example) (Cullen et al., 2019). Similarly, quantifying physiological or pathological markers inside cerebral organoid regions has been performed only with semi-automatic tools (Anastasaki et al., 2020; Jacob et al., 2020; Qian et al., 2020; Zhang et al., 2020). Automatic tools developed for other culture models could potentially be used to achieve such quantification (Piccinini et al., 2020). Development of new methods could also be inspired by approaches already used for *in vivo* brain models (Zhang, 2017), however with some limitations regarding methods used for real human brain tissue study. In this specific case, cell counting is based on three different approaches: histological or stereological approaches, DNA extraction, and isotropic fractionating. Only the first method keeps the localization of the cells (von Bartheld et al., 2017) and would therefore be suited for cerebral organoids.

Another interesting project to develop is the creation of cerebral organoid connectomes. We think connectivity mapping has to be developed at various scales, between two organoids of an assembloid, between regions inside an organoid, but also between the constituting cells. In assembloids, the connectivity could help to explain neurodevelopmental defects using pluripotent stem cells derived from neurological diseases patients and to test potential therapeutic compounds (Bagley et al., 2017). Another review addresses the challenge of connecting an organ culture with cerebral organoids in order to reproduce important axes in the human body, although this raises major ethical questions (Chukwurah et al., 2019).

New computational methods identifying connections could help to understand organoid inner structure. For instance, regional connectivity could be helpful to identify a pathological formation inside the neuroepithelium, and help to understand the neurodevelopmental formation (Seto and Eiraku, 2019). Finally, characterizing the full connectivity of the whole organoid, or inside a particular region, could help distinguishing relations between different cell types, relevant to identify neurodegenerative diseases (Marotta et al., 2020).

**Figure 7:**
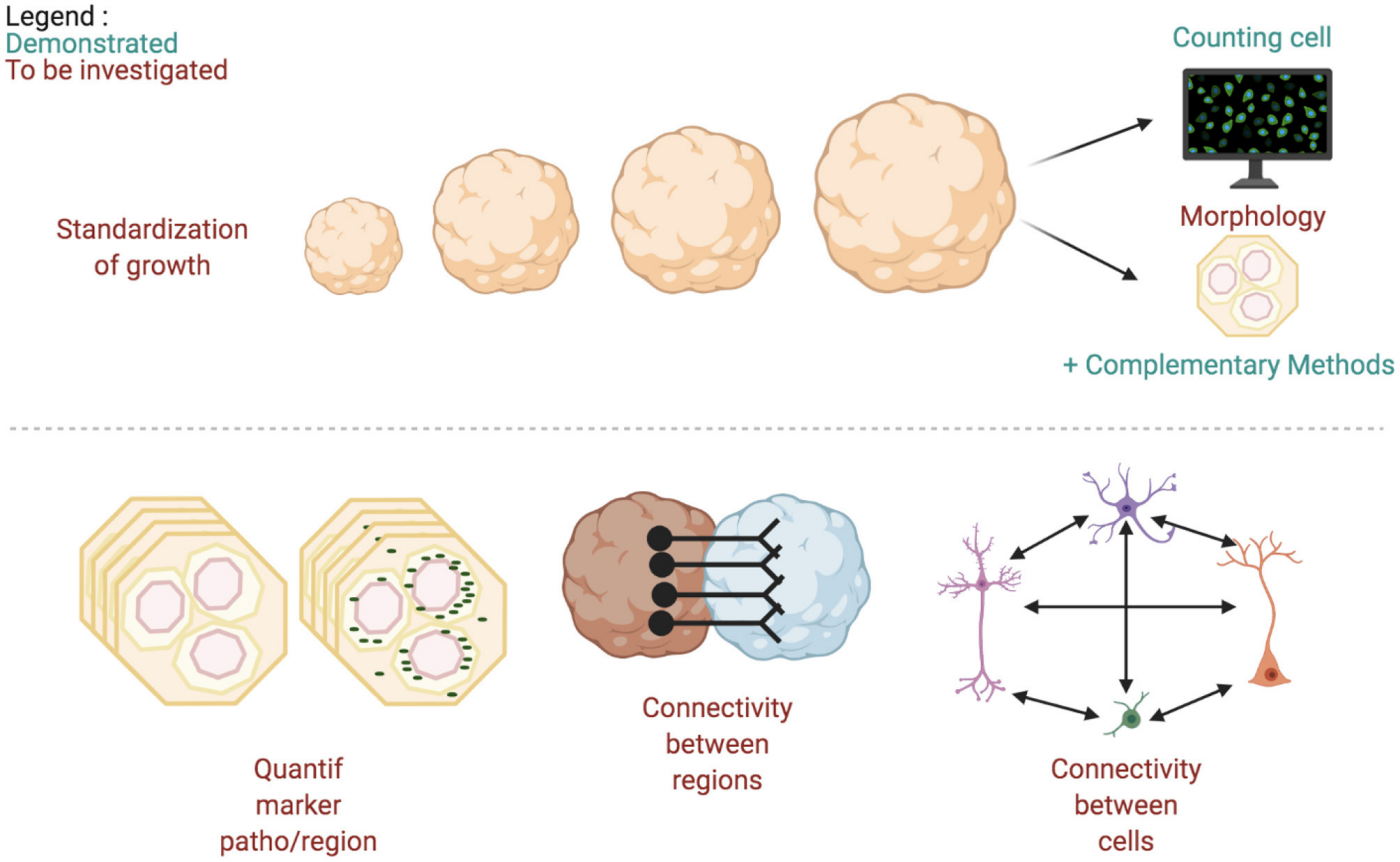
Quantitative and morphological expected tools to analyze images of 3D brain cultures.

The identification of cell interactions has been described in another review (Poli et al., 2019), and is based on a connectivity map realized after segmentation of clarified tissues and visualized with virtual reality. This method could also be applied for fused regional cerebral organoids or for connected organ culture with the brain one. A unified analysis tools platform would benefit simultaneously to the manufacturing process standardization and 3D cultures research (summarized in Figure 7).

## 7. Conclusion

The use of cerebral organoids in laboratories has increased exponentially since their first creation in 2013. However, we observe in this review that actual tools to study images from these 3D brain cultures in all their dimensions suffer from some limitations. The structural variability occurring during maturation needs to be limited by improved protocols or by computational analysis solutions. The best combination of "clarification protocol–microscopic device" remains to be highlighted to acquire images from cerebral organoids that could be analyzed in all their dimensions. Specific tools need to be developed to improve the speed and the accuracy of their identification and quantification, but also to better understand their physiology and their entire 3D cyto-architecture. However, such an approach implies access to very large image datasets, which seems only possible when they will be stored in the “Organobanks.”

As already mentioned by two other teams, and once the current limitations are overcome, the ideal platform would combine molecular/transcriptome and high-throughput image analysis tools (Poli et al., 2019; Grenier et al., 2020). The first milestone of this kind of research was very recently published (Albanese et al., 2020; Renner et al., 2020). However, cerebral organoids dedicated image analysis tools remain to be developed, as summarized in Figure 7.

We are convinced that cerebral organoids coupled with high-performance image analysis tools have the potential to highlight features that other brain models are not able to show yet, and will help evaluating theories in the neuroscience field.

## Author Contributions

This review article is an idea of AH. The literature search and data analysis was performed by CB. Graphics tables and figures were conceived by CB and CS. The first draft was written by CB while CS, CC, and AH critically revised the work. All authors contributed to the article and approved the submitted version.

## Conflict of Interest

The authors declare that the research was conducted in the absence of any commercial or financial relationships that could be construed as a potential conflict of interest.

## Footnotes

^1^MathWorks, Matlab TM.

^2^https://software.rc.fas.harvard.edu/lichtman/vast/.

^3^https://www.visiopharm.com/.

^4^https://www.leicabiosystems.com/fr/imagerie-pathologique/analyser/.
